# Feasibility and safety of total laparoscopic hysterectomy for huge uteri without the use of uterine manipulator: description of emblematic cases

**DOI:** 10.1186/s10397-018-1037-5

**Published:** 2018-02-26

**Authors:** Antonio Macciò, Clelia Madeddu, Paraskevas Kotsonis, Giacomo Chiappe, Fabrizio Lavra, Ivan Collu, Roberto Demontis

**Affiliations:** 1 Department of Gynecologic Oncology, Azienda Ospedaliera Brotzu, via Jenner, 09100 Cagliari, Italy; 2 Department of Medical Sciences and Public Health, University of Cagliari, Cagliari, Italy; 3 Department of Medical Sciences and Public Health, Section of Forensic Medicine, University of Cagliari, Cagliari, Italy

**Keywords:** Uterine fibromatosis, Total laparoscopic hysterectomy, Huge uteri, Uterine manipulator

## Abstract

**Background:**

Uterine manipulator is a very useful tool in performing total laparoscopic hysterectomy (TLH) for large uteri; however, in some cases, it cannot be used due to unfavorable anatomical conditions. The feasibility and safety of TLH for very large uteri without the use of uterine manipulator has not yet been established.

**Results:**

We describe two emblematic cases of TLH for huge fibromatous uteri: the first one for a uterus weighing 5700 g, which is the largest uterus laparoscopically removed to date reported in literature, and the second one for a uterus of 3670 g associated with a severe lymph node neoplastic disease.

In both cases, TLH was successfully and safely performed even without the use of uterine manipulator, thus allowing a rapid recovery, especially in the second case, which was essential for a fast start of the most appropriate oncological treatment, the best quality of life and undoubtedly cosmetic advantages.

**Conclusions:**

Although we believe in the great usefulness of the uterine manipulator in performing TLH for huge uteri, in the present paper, we demonstrate the feasibility and safety of such complex surgery also when the use of this tool is not possible due to unfavorable anatomical condition.

## Background

Hysterectomy is one of the most commonly performed gynecological procedures. Since the first total laparoscopic hysterectomy (TLH) has been published in 1993 [1], surgeons have tried to identify tools and techniques that could make surgery simpler and safer. Uterine manipulators were among the first instruments introduced to improve laparoscopic performance [2]. Indeed, to date, most publications state that manipulators provide multifunctional assistance in gynecologic surgery, particularly during TLH [3]. Recently, we emphasized the usefulness of the uterine manipulator for laparoscopic removal of large fibromatous uteri ranging from 300 to 5320 g [4, 5]. We clarified that the manipulator aids in mobilizing the uterus to better define surrounding organs to display the vaginal fornices for easier culdotomy and to move the ureter from the uterine cervix to avoid damage. However, there are situations where, for anatomical reasons, the uterine manipulator cannot be used; for example, in the case of vaginal stenosis or other anatomic situations in which it is difficult to identify the uterine cervix. We believe that such situations should not prevent the use of laparoscopic surgery, even in the case of very large fibromatous uteri, and that these issues should be adequately explored and discussed. For this reason, we describe two emblematic cases of laparoscopic removal of a huge fibromatous uterus weighing 5700 and 3670 g, respectively, without the use of a uterine manipulator.

## Methods

The TLH was performed according to the procedure we previously reported [4, 5]. However, in this case, we omitted the use of the uterine manipulator due to unfavorable anatomical findings. A 12-mm trocar was positioned via an open entry technique nearly the xiphoid process, and a 10–14-mmHg pneumoperitoneum was obtained. Four 5-mm trocars were positioned laterally to the rectus abdominis; the lowest of these was used, in the absence of the uterine manipulator, to push cephalad the markedly enlarged uterus to reproduce the “traction-counter traction” effect and obtain both the displacement of the lower uterine segment aside from ureters and elevation of uterine arteries alongside the cervix. A 12-mm trocar was placed at the umbilicus, and another 5-mm trocar was inserted in the suprapubic position. The surgery table was positioned in the Trendelenburg orientation, and the stages of surgery were as previously described by the authors [5] for the removal of uterus of similar weight combined with bilateral adnexectomy, using Ligasure (Tyco Healthcare, Norwalk, CT, USA). The uterine arteries were skeletonized, coagulated with the BiClamp LAP forceps (ERBE GmbH, Tubingen, Germany), and then resected with Ligasure. After the introduction of a small surgical swab in the vagina, the cervicovaginal edge was laparoscopically identified and catted with monopolar scissor, and the colpotomy was completed using Ligasure. As is our routine practice, after the cervix dissection, a Foley catheter was inserted into the vagina to prevent losing of the pneumoperitoneum. The laparoscopic suture of the vaginal cuff was performed with a continuous closure using the V-Loc device (Covidien-Medtronic, Minneapolis, MN, USA). The uterus was then extracted from the abdomen via a very low transverse laparotomy cut of approximately 5 cm, to reduce operative time, utilizing a wound protector/retractor (Wound Edge Protector - 3MTM Steri-DrapeTM 1073, Diegem, Belgium), and morcellated outside the abdomen with a cold blade scalpel to avoid spillage. Finally, after suture of the low minilaparotomy, we laparoscopically cautiously evaluated the abdominal cavity and repeatedly washed it.

## Results

### Case 1

**Fig. 1:**
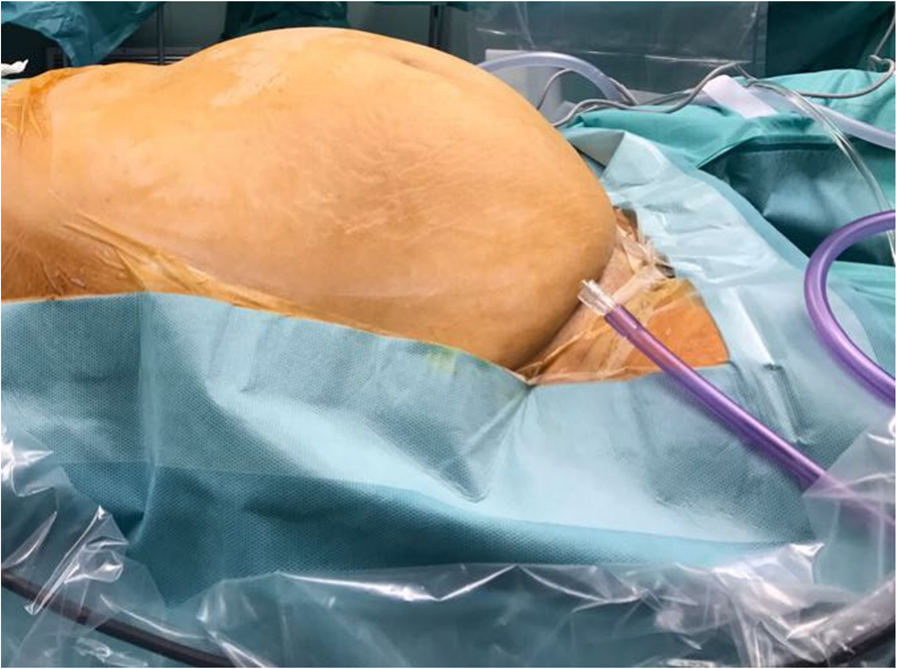
Case 1: enlarged abdomen with the patient lying supine before surgery

A 52-year-old, Caucasian, 1 para, obese (body mass index (BMI) 32.46) female presented to our department for progressive increase in abdominal circumference in the previous year associated with constipation and dyspnea. A fibromatous uterus had been diagnosed previously by pelvic magnetic resonance. Her surgical history included a previous cesarean section. No relevant disturbances of the menstrual cycle were referred by the patient. Physical exam showed an abdomen entirely occupied by a pelvic mass reaching the xiphoid process, which was especially evident when the patient laid supine (Fig. 1). On bimanual pelvic examination, the superior third of the vagina was making it impossible to visualize the cervix. Abdominal and vaginal ultrasound performed on hospital admission confirmed a huge fibromatous uterus. Cervicovaginal smear and endometrial sampling, to exclude potential endometrial cancer, could not be performed for the above reported anatomical reasons. The patient had normal hematological, the liver and renal function parameters. Tumor markers were within the normal range. The patient was counseled on the various surgical options and the associated risks, and she opted for a minimally invasive approach, if feasible. Then, detailed written informed consent, prepared by a forensic expert physician, was obtained for the procedure as well as for the publication of a case report and the accompanying images. The TLH was performed as described above. No intraoperative complications occurred; the operative time was approximately 200 min. Intraoperative blood loss was 300 ml due to bleeding at the time of skeletonization of the left uterine vein, which was particularly large and frail. The removed uterus weighed 5700 g. The histological examination revealed a benign fibroid uterus. The patient left the hospital on postoperative day 3 in a very good state. Seven days after discharge she was readmitted to our department because of fever with elevated C-reactive protein (CRP) level and white cell count associated with left basal thoracic pain; then, she underwent total body computed tomography (CT) that showed basal bronchopneumonitis, which resolved with antibiotics. The patient was discharged after 2 days and continued antibiotic therapy at home. One month after discharge, the patient was in excellent condition.

### Case 2

**Fig. 2:**
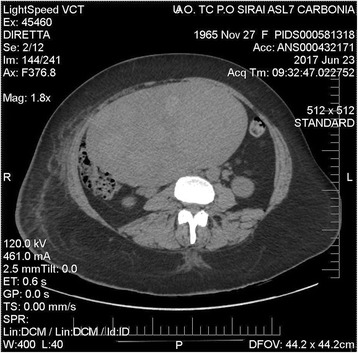
Case 2: axial computed tomography scan of the abdomen showing a huge inhomogenous uterus measuring more than 20 cm occupying entirely the right side of the pelvis

**Fig. 3:**
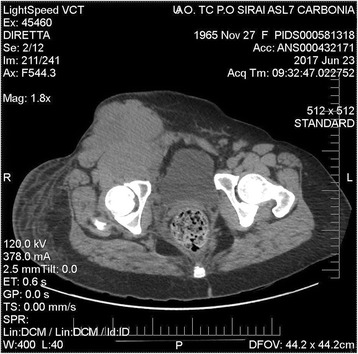
Case 2: axial computed tomography scan showing the lymph node mass (14 × 15 cm) extending to the origin of the right thigh

A 50-year-old obese (BMI 34.25) woman was referred to our department from another hospital because we are the Regional Referral Center for cancer disease, with the diagnosis of a huge abdominal mass and a large right pelvic lymph node mass associated with severe edematous enlargement of the ipsilateral lower limb, suspicious for thrombophlebitis. On bimanual pelvic examination, the vagina was occupied by a voluminous spherical mass, the size of a tennis ball. The mass originated from the right vaginal fornix, making it impossible to reach and evaluate the cervix. A large fibromatous mass, reaching the third space above the transverse umbilical line, filled entirely the abdomen. Total body CT showed a huge inhomogeneous uterus measuring more than 20 cm, occupying entirely the right pelvis (Fig. 2). This lesion appeared in continuity with another inhomogeneous mass, considered likely to be lymphatic in origin, measuring approximately 14 × 15 cm, extending to the proximal extremity of the right thigh (Fig. 3), and incorporating the vascular structures from the aortic bifurcation to the iliac-femoral venous axis. The large abdominal mass at the right iliac fossa constricted the vena cava, the bladder, and the right ureter with initial pyeloureteral dilatation. No pelvic effusion nor pathological findings outside the abdomen and pelvis were noted. On admission, laboratory analyses showed high levels of CA-125 (2195 U/ml, normal level < 35), C-reactive protein (0.3 mg/dL, normal range 0–0.10), fibrinogen (448 mg/dL, normal range 200–400), LDH (1976 U/L, normal range 0–248), and low hemoglobin (10.5 g/dl, range 12–15), iron (32 mg/dL, normal range 60–180), and ferritin (34 ng/mL, normal range 10–291). Assuming the presence of two pathologies, i.e., a massive fibromatous uterus and a myeloproliferative disease associated with thrombophlebitis of the right leg due to compression, we proposed that the patient underwent a mini-invasive exploratory laparoscopy with possible hysterectomy plus bilateral adnexectomy and lymph node biopsy. This approach was chosen based on the urgent need to decompress the pelvis from the massive uterine mass and to reduce the patient’s delay in receiving the appropriate chemotherapy. The patient signed an informed consent and agreed to our surgical plan. The surgical technique was as described in the previous section, without the use of the uterine manipulator due to the unfavorable findings of the vagina occupied by the massive lymph node masses. In particular, the ureter isolation was required throughout its course, freeing it from the adhesions with the massive lymph nodes extending from the origin of the common iliac artery to the inguinal canal, including the hypogastric artery. Both uterine arteries were coagulated at the origin of the hypogastric artery. The surgery was subsequently carried out as reported above, with the support of a laparotomy incision of approximately 5 cm. Prior to the removal of the uterus, an extensive excision of the lymph node mass was performed at the level of the common right iliac artery, and the specimen was sent for intraoperative histological examination, with a diagnosis of lymphoproliferative disease. The definitive histology diagnosed a large B cell lymphoma, present in 20 out of 24 removed lymph nodes, with an ovarian secondary involvement. The postoperative course was optimal and the patient was quickly transferred to the Department of Oncology/Hematology at our Institute, where she is currently receiving the appropriate chemotherapy treatment.

## Discussion

The central role that laparoscopy now plays in gynecological surgery is indisputable, especially in the case of TLH. A recent Cochrane analysis has reported that vaginal hysterectomy (VH) seems to be a superior minimal access method for benign gynecological disease in comparison to TLH, although the authors emphasized in their conclusions that the surgical approach to hysterectomy should be decided with the patient on the basis of the relative benefit and risks, which are dependent on the surgical expertise [6]. In fact, it must be clarified that, despite the widespread use of laparoscopic hysterectomy, this approach should be applied in those cases where VH cannot be performed or would be much more complex, or in case of adnexal disease or where laparoscopy avoids open surgery while offering certain advantages in efficacy, safety, time of hospitalization, and recovery. It should be added that, among the different procedures, TLH has higher costs in comparison to the vaginal and abdominal surgery [7]. Then, the comparison between various surgical procedures can be effectively performed only when the indications for surgery are similar, clear, and appropriate. The choice of surgical approach in the case of a large uterus presents an entirely different discussion. The precise surgical conditions that make the TLH technique for large uteri feasible, repeatable, and safe have been codified so that skilled and well-coordinated teams choose it as the first choice approach rather than laparotomy, even in the case of huge uteri. The publications of these working groups, including the current authors, have highlighted some central points regarding the performance of TLH for very large uteri, including pneumoperitoneum created in the neutral position, trocar number and location dependent upon the uterine size, modifying the trocar positions during the surgery for a better view, and the uterine vessel coagulation using the BiClamp to provide very good hemostasis [4, 8, 9]. With these two clinical cases, we describe the feasibility of performing TLH without the use of the uterine manipulator, even in the case of very large uteri. Such technical possibility has been already described [10], but for uteri of a size not comparable to the cases herein reported. Far from affirming the usefulness of the uterine manipulator, however, we demonstrated the feasibility and safety of such complex interventions even in its absence. We emphasize that only unfavorable conditions should lead to perform this surgery without this valuable tool. Moreover, we believe that the description of these interventions and their technical modalities should be a matter of discussion and may aid those surgeons who choose this mini-invasive approach. The unique clinical features of our two cases should also be highlighted. In the first case, the uterus was extremely great, weighing 5700 g, similar to a case we previously reported of 5320 g [5]; this, therefore, constitutes the description of the largest uterus removed laparoscopically in the published literature to date. The second case is notable for the association of a massive fibromatous uterus with severe lymph node neoplastic disease, which created difficulty in providing an exact diagnosis and also in choosing the most appropriate surgical techniques for a successful outcome. In this second case, our approach allowed the patient’s rapid healing and fast initiation of oncologic treatment.

## Conclusions

These peculiar clinical cases reaffirm the efficacy and safety of TLH, even in the case of very large uteri, also without using an important instrument such as the uterine manipulator. The presence of other associated pathologies (i.e., the pelvic lymph node disease), while making the intervention more complex and lengthy, underlines that, also in such truly complex cases, TLH is a valid alternative to open surgery, provided that highly experienced and well-coordinated surgical teams perform it. The immediate recovery after TLH is also a major aspect in the patient comprehensive clinical management, as in the second case herein described where the patient was able to access the most appropriate antiblastic therapy as soon as possible in excellent postoperative conditions and, thus, with great therapeutic benefits.

## Abbreviations

BMI: Body mass index
CT: Computed tomography
TLH: Total laparoscopic hysterectomy
